# The fight against fraud continues

**DOI:** 10.1002/nop2.189

**Published:** 2018-07-23

**Authors:** Roger Watson

I received the following email from a colleague (edited slightly):

I am writing to seek advice for my friend. He was a victim of a hijacked journal. The name of the journal is XXXXXXXX Review. He paid US$600 as an APC. He later learned that the journal is a hijacked journal. He has emailed the journal requesting to retract the paper and to possibly refund the publication fee. However, until now, he got no response

and he asked if there was anything that could be done. My response was a curt “no.” Apparently, he had tried to contact the journal to no avail.

What is illustrated above is one of the many aspects of the “hydra‐headed monster” of the predatory publishing “industry.” In this case, the unfortunate friend of my colleague had submitted to a website — probably responding to a predatory email — that appeared to be convincing and possibly identical to a genuine journal website. However, the journal identity had been used to set up a nearly identical website or an old version of the website had been found on the Internet and reactivated. This kind of criminal activity goes beyond even the questionable practices of most predatory journals. These, at least in most circumstances, publish your paper albeit without a peer review process, no editing and the minimum of production. In the case of a hijacked journal website, your paper will never be published, and you will have paid for nothing. The rise of these hijacked websites was first brought to my attention in the editorial pages of *Journal of Advanced Nursing* (Dadkhah, 2016).

What can you do to protect yourself from the hijacked websites? First, never respond to any emails asking you to submit a manuscript to a journal. Reputable journals never send out such invitations and especially never attempt the kind of ridiculous flattery: “greetings”; “esteemed professor”; “hoping you are in good health”; “we await your manuscript”; “we need one more to complete our next volume”; or any nonsense like that. On a desktop or portable computer simply delete; on a smartphone…swipe to the left! If you plan to submit and find a journal website yourself, you are on very safe ground. Nevertheless, you may be directed to a hijacked website and it may be hard to tell. If you have any suspicions, then try searching for the website again and compare with what you find. Check URLs carefully — is the URL that of a reputable publisher — e.g., Wiley — which will provide the platform for the journal? If in doubt, email or phone the journal and get the URL directly from them. If you do proceed with a hijacked website, you will inevitably be alerted at the point where payment is requested. Note carefully — no reputable journal, including open access journals such as *Nursing Open*, will request money at the point of submission. Payment is only requested if your manuscript is accepted. If you reach a point where payment is requested on submission, retreat and report the website to the genuine publisher of the journal you are targeting. If in doubt; don't!
